# Prognostic impact of ICG-PDR in patients with hypoxic hepatitis at the intensive care unit

**DOI:** 10.1186/2197-425X-3-S1-A974

**Published:** 2015-10-01

**Authors:** T Horvatits, N Kneidinger, A Drolz, K Roedl, K Rutter, S Kluge, V Fuhrmann

**Affiliations:** Department of Intensive Care Medicine, University Medical Center Hamburg-Eppendorf, Hamburg, Germany

## Introduction

Hypoxic hepatitis (HH) is a frequent cause of acute hepatocellular damage in critically ill patients associated with high mortality. Indocyanine green, a medical dye, is removed solely by the liver without entering enterohepatic circulation. Therefore its plasma disappearance rate (ICG-PDR) is an effective clinical tool for assessment of liver function in acute and chronic hepatic disease.

## Objectives

Aim of this study was to evaluate the prognostic impact of ICG-PDR in comparison to laboratory parameters and established scores in critically ill patients with HH and other liver disease entities.

## Methods

The impact of ICG-PDR was prospectively evaluated in 119 critically ill patients with different liver disease entities (HH: n = 52, cirrhosis: n = 35, acute liver failure (ALF): n = 10, control group without signs of acute liver injury, viral or drug induced hepatitis or cirrhosis: n = 22). Plasma disappearance rate of ICG was measured non-invasively by using a finger pulse densitometry system.

## Results

ICG-PDR on admission was significantly lower in patients with liver diseases (HH, liver cirrhosis, ALF) than in the control group without hepatic impairment (median 5.7%/min, IQR 3.8-7.9%/min vs. 20.7%/min, IQR 14.1-25.4%/min; p < 0.001).

ICG-PDR predicted 28-day mortality independently of SOFA score and serum lactate in patients with underlying liver disease (HR 1.27, 95%CI 1.10-1.45; p < 0.005). AUROC of ICG-PDR predicting 28-day mortality in patients with HH was significantly higher than AUROC of SOFA, arterial serum lactate, INR and AST 48 hours after admission (p < 0.05). ICG-PDR increased significantly in 28-day survivors over course of time (p < 0.001). 28-day survivors could be identified using a cut-off ICG-PDR ≥ 9.0 %/min 48 hours after admission with sensitivity of 77% and specificity 100%.

## Conclusion

Diagnostic test accuracy of ICG-PDR was superior to standard liver function parameters and well established scoring systems in patients with HH. Therefore ICG-PDR is a feasible non-invasive tool for early risk stratification in patients with HH.

